# Fine motor deficits and attention deficit hyperactivity disorder in primary school children

**DOI:** 10.4102/sajpsychiatry.v25i0.1232

**Published:** 2019-01-22

**Authors:** Maria Mokobane, Basil J. Pillay, Anneke Meyer

**Affiliations:** 1 Department of Behavioural Medicine, University of KwaZulu-Natal, South Africa

## Abstract

**Background:**

Many children with attention deficit hyperactivity disorder (ADHD) display motor deficiencies during their daily routine, which may have impact on their developmental course. Children with ADHD who experience motor deficiencies often display deficits in tasks requiring movements, such as handwriting.

**Aim:**

This study investigated deficiencies in fine motor skills in primary school children with ADHD. The study further sought to establish whether ADHD subtypes differ in deficiencies of fine motor performance, recorded for both the dominant and non-dominant hands.

**Methods:**

The Disruptive Behavior Disorders Rating Scale, completed by educators and parents, was used to screen for ADHD symptoms. Researchers confirmed the diagnosis of ADHD. Motor functioning was assessed using the Grooved Pegboard and Maze Coordination. The children diagnosed with ADHD were matched for age and gender with controls without ADHD. The sample consisted of an ADHD group (160) and control group (160) of primary school children from the Moletjie area.

**Results:**

Children with ADHD (predominantly inattentive subtype) and ADHD (combined subtype) performed significantly more poorly than the control group on the Grooved Pegboard (*p* < 0.05) with both the dominant and non-dominant hand. No significant difference between the hyperactivity and impulsiveness subtype and the controls were found. There was no difference on the Maze Coordination Task (*p* > 0.05) between the ADHD subtypes and the controls.

**Conclusion:**

Difficulties in fine motor skills are prevalent in children with ADHD, particularly in the ADHD-PI and ADHD-C. Problems are encountered in distal, complex, speeded tasks. The effect may lead to poor handwriting and academic performance.

## Introduction

Attention deficit hyperactivity disorder (ADHD) is a common, long-lasting, manageable childhood psychiatric disorder, characterised by a pattern of developmentally inappropriate inattention, motor restlessness and impulsiveness.^1^ It affects approximately 3% – 7% of school-aged children worldwide^1^ and is characterised by symptoms of severe inattention, impulsiveness and overactivity.^2^ Poor motor coordination or motor performance is another common coexisting difficulty in children with ADHD, though it has received less attention in research.^3^ Children with ADHD who experience motor difficulties often display deficits in tasks requiring coordination of complex movements, such as handwriting. Such children often appear clumsy and uncoordinated.^4,5^ Motor coordination problems have previously been labelled ‘clumsy child syndrome’, ‘non-cerebral-palsy motor-perception dysfunction’, ‘minor neurological dysfunction’ or ‘dyspraxia of childhood’.^6^

Motor skills are actions carried out when the brain, nervous system and muscles work together. It is a function that involves the precise movement of muscles with the intent to perform a specific act. They are categorised into two groups: gross motor skills and fine motor skills. Gross motor skills are involved in movement and coordination of the arms and legs and actions such as running, crawling and swimming.^7^ Fine motor skills are required in smaller movements that occur in the wrists, hands, fingers, feet and toes and include more precise actions such as picking up objects between the thumb and finger and writing carefully.^8^ Poor fine motor skills can make cognitive learning and performance more difficult because of the involvement of fine motor skills in cognitive activities.^5,8^

Since 1994, the use of the term ‘developmental coordination disorder’ (DCD) has predominated in the literature. In the Scandinavian countries, the combination of ADHD and motor coordination problems is known as ‘deficits of attention and motor perception’ (DAMP).^9,10^ Recently it was suggested that the name ‘DAMP’ be changed to ‘DCD plus’.^11^ Developmental coordination disorder is diagnosed if the impairment in motor skills significantly interferes with the performance of, or participation in, daily activities in family, social, school or community life.^2^ Developmental coordination disorder does not have separate classifications; however, individuals may be impaired predominantly in either gross or fine motor skills, including handwriting skills. The core characteristic of DCD involves a marked impairment in the performance of motor skills. This impairment has a negative impact on activities of daily life such as dressing, feeding and riding a bicycle or academic achievement through poor handwriting skills. DCD is found in up to 50% of the children with ADHD.^3,12^

Many of the differences found in the neural systems between ADHD and neurotypical comparisons are present in the areas responsible for motor control.^13,14^ Kaiser et al.^15^ argue that individuals with DCD and ADHD may fall, bump into things or knock things over; this may not be because of distractibility and impulsiveness but rather because of motor impairment. Children with ADHD tend to show persistent motor skill impairment, which might meet the diagnostic criteria of DCD as a comorbid disorder.^15^

The prevalence of motor problems in children with ADHD ranges from 30% to 52%, depending on the method of measurement.^16,17^ A study by Pitcher et al.^18^ found that 58% of children with ADHD (predominantly inattentive subtype, (ADHD-PI), 49% with ADHD combined (ADHD-C) and 47% with ADHD hyperactivity and impulsiveness (ADHD-HI) had motor problems. In clinical practice, less attention is paid to motor problems in children with ADHD.^19^

Most studies show a strong association between ADHD and fine motor problems.^18,20^ Kadesjö and Gillberg^9^ and Piek et al.^21^ affirm that inattentive symptoms relate mostly to motor coordination problems, though a relationship between hyperactive and impulsive symptoms and motor coordination problems has also been reported. Motor problems lead to difficulties in everyday living, including academic performance, sport, play and self-esteem.^1,21,22,23^ Motor problems severely affect children’s daily lives and are an active predictor of a child’s popularity and self-esteem.^24^ These deficits may have an intense effect on children’s development, leading to difficulty with written communication, inhibited social interaction and poor performance in sports activities.

Several studies have identified dopamine as the key neurotransmitter in the brain in ADHD. Three pathways, namely the mesocortical, mesolimbic and nigrostriatal, have been shown to be dysfunctional and consequently cause deficiencies in the cortical areas, resulting in symptoms typical to ADHD: inattention, hyperactivity and impulsiveness.^25,26,27^ The nigrostriatal dopaminergic pathway is involved in the coordination of movement. Sagvolden et al.^26^ assert that a hypofunctioning nigrostriatal dopamine branch causes impaired modulation of motor functions and deficient non-declarative habit learning and memory. The altered dopaminergic function and hypofunctioning nigrostriatal dopamine will give rise to clumsiness and problems with gait, balance and laterality, as well as gross and fine motor control.^26^ Difficulties with motor inhibition may be associated with disturbances in the orbital prefrontal circuit system, which also plays a central role in executive functions.^28^ This view is further supported by Berquin et al.,^29^ who suggest that cerebellar-prefrontal circuit dysfunction may underlie the motor control, inhibition and executive function deficits encountered in ADHD.

Motor problems are not usually part of assessments for ADHD and are not included in intervention programmes^10,30^ despite the estimate that 30% – 50% of children with ADHD exhibit motor problems.^3^ This study aimed to investigate fine motor difficulties in a sample of primary school children diagnosed with ADHD in Limpopo Province, South Africa. The study further sought to establish whether ADHD subtypes or presentations differ in motor performance deficiencies, whether this is true for both the dominant and non-dominant hand, and whether gender and age influence performance. Understanding the defects in fine motor skills and how the ADHD subtypes are affected will enable clinicians, educators and parents to devise appropriate intervention methods in the treatment of children with ADHD. The results will also help them to better understand the nature of fine motor deficits in children with ADHD to inform and guide future treatment and therapy for these children.

## Research methods and design

### Study design

The study was conducted in primary schools in the Moletjie area, Limpopo, in 2016. A quantitative, case-control experimental design was used. In order to establish whether children with ADHD are deficient in fine motor skills, the sample was divided into participants diagnosed with ADHD and those without ADHD.

### Setting

The study was conducted with primary school children in the Moletjie area in Limpopo Province, South Africa. The selected regions were in a rural area in which assessment of this nature is very rare. The areas were selected based on the remoteness of the regions so that the community could benefit from such research.

### Study population and sampling strategy

Four thousand two hundred children completed the Disruptive Behavior Disorders (DBD) Rating Scale. A total of 320 primary school children were screened; 160 with ADHD (80 boys and 80 girls) were matched for gender and age with 160 children without ADHD symptomatology (80 boys and 80 girls), who formed the control group. They were all Sepedi speaking, Grade 1 to Grade 7 learners, between the ages of 6 and 14 years, and were recruited from six schools in the Moletjie area, Limpopo Province, South Africa. The DBD, completed by educators and parents, was used to screen the children for ADHD.^31,32^ Learners who met the criteria for ADHD were assigned by the researcher as follows: Participants with scores ≥ 17 on the hyperactivity and impulsiveness scale were classified as ADHD-HI subtype and those having a score ≥ 20 on the inattention scale were classified as ADHD-PI subtype, based on the epidemiological study by Meyer and colleagues.^33^ Participants who met the criteria on both scales were categorised as ADHD-C subtype. The cut-off point for the neurotypical control group (non-ADHD) was set at the 85th percentile or below to decrease the risk for false positives in this group.^33^ Thus, children with scores of less than 15 on the hyperactivity and impulsiveness scale and the inattention scale, matched for gender and age, were selected as controls.

Children with a history of neurological problems (e.g. head injuries, epilepsy, cerebral palsy, etc.) or severe psychiatric disorders, as reported by the parents on the demographic questionnaire, were excluded from the study. None of the children were on psychostimulant medication at the time of testing.

### Data collection

The principals of the schools were approached prior to commencing with the assessment. Following consent from parents, the study was explained to all participating children, and their assent was obtained. The return rate of the questionnaires was 100%. The data was collected from primary school children within the Moletjie area in Limpopo Province during 2016. The assessment was done during school hours at the participants’ respective schools. The testing procedure for each child lasted 30 min and was conducted by a clinical psychologist and five research assistants (who held bachelor degrees in psychology), who were fluent in the child’s home language.

### Instruments

#### Screening instruments: Disruptive Behavior Disorders Rating Scale

The DBD Rating Scale^31,32^ was used to screen for ADHD symptoms. The scale is standardised and normed for all language and population groups in Limpopo Province, South Africa.^33^ The DBD assesses the presence and the degree of ADHD-related symptoms (inattention and hyperactivity/impulsiveness), oppositional defiant disorder and conduct disorder.^34^ The DBD consists of 42 items based on the diagnostic statistical manual of mental disorder (DSM-IV) diagnostic criteria,^35^ of which 18 measure ADHD. All 42 items were administered. Respondents were asked to rate the behaviour on a four-point scale: not at all (0); just a little (1), pretty much (2) and very much (3). The scores were summed to produce a total score, and a 95th-percentile cut-off score is considered clinically significant.^28^ Those participants scoring below the 85th percentile formed the control group. The Cronbach’s alpha for the DBD for the targeted population was calculated at 0.90 for the hyperactivity and impulsiveness scale and 0.92 for the inattention scale.^33^

#### Assessment of motor functions

The Grooved Pegboard, measuring distal, complex fine motor coordination and psychomotor speed, was administered first, followed by the Maze Coordination Task, which evaluates tactual motor coordination skills and motor planning.^36^

#### Grooved Pegboard Task

The Grooved Pegboard is a manual dexterity test measuring complex fine motor skills.^36^ This task consists of a small (10 cm × 10 cm) metal board that contains a 5 × 5 set of holes, each with a groove, oriented randomly in different directions. Twenty-five round metal pegs with a ridge running lengthwise have to be rotated to the correct position for insertion into the holes. The participants are instructed to insert the pegs as quickly as possible into the slots in sequence, first with the dominant hand and then with the non-dominant hand. The hand that they use to write was recognised as the dominant hand; the one not being used was recognised as the non-dominant. Pegs are inserted from *left to right with the right hand and vice versa for the left*. The score is the time it takes the participant to complete the task with each hand. The task duration is approximately 5 min.^37^ The Grooved Pegboard has good test reliability (Cronbach’s α 0.91 and 0.85 for right and left hands, respectively). The Grooved Pegboard correlates with the Bruininks-Oseretsky Test at −0.50 to −0.63 and with the Purdue Pegboard at −0.73 to −0.78.^38^ Studies^21,39,40^ have shown that the Grooved Pegboard could efficiently be used to detect problems with fine motor skills in children with symptoms of ADHD.

#### Maze Coordination Task

The Maze Coordination Task measures fine motor coordination.^39^ The Maze is placed at a ~60° angle to the table. The child is required to go through the maze with an electric stylus, trying not to touch the sides. The stylus is connected to an electronic clock and a counter, which record the number of contacts the stylus is making with the sides (counter) and the cumulative duration of these contacts (timer). The aim is to move the stylus through the maze without touching the sides. There is no speed requirement, and the test is performed twice with each hand. The total sum of touches and cumulative time of contact of two trials with the same-side hand are the final scores. According to Tichá et al.^41^ and Torgesen et al.,^42^ reliabilities range from 0.27 to 0.91.

### Data analysis

Statistica (version 10)^43^ was used to analyse data from the tests mentioned above. Multivariate analysis of variance (MANOVA) models were used to determine differences in performance. A 4 × 2 × 3 (ADHD presentation × gender × age group) analysis of variance for independent samples for both the dominant and the non-dominant hand were computed to establish between-group differences. Post-hoc tests (Bonferroni) were used to establish within-group differences. The significance value was set at *p* < 0.05.

## Ethical consideration

I am enclosing a submission to the *South African Journal of Psychiatry*. The manuscript is the original and was never presented to any other journal and my institution, the University of KwaZulu-Natal, is fully aware of this submission. Ethical approval to conduct the study was obtained (Ethical clearance number: HSS/0702/015D) from the Biomedical Research Ethics Committee of the University of KwaZulu-Natal. The Department of Education provided permission for the study to be conducted at the participating schools, and consent was obtained from the relevant school principals. Written consent was obtained from the parents of the children, and children signed assent forms. Participation in the study was voluntary, and participants were informed that they could withdraw at any stage.

## Results

A total of 4200 children completed the DBD, of which 320 children were screened in the study: 160 (50%) males and 160 (50%) females. One hundred sixty children (80 boys and 80 girls) met the criteria for ADHD (*n* = 160) and were matched for age and gender with a control group (*n* = 160) without ADHD symptomatology. The equal numbers in gender was coincidental. There was no statistically significant difference in ages of the ADHD group (*M* = 10.52, s.d. = 1.76) and control group (*M* =10.49, s.d. = 1.80, *p* = 0.94).

The grade distribution was as follows: Grade 1 – ADHD = 8, control = 8; Grade 2 – ADHD = 14; control = 13; Grade 3 – ADHD = 26, control = 24; Grade 4 – ADHD = 37, control = 33; Grade 5 – ADHD = 41, control = 39; Grade 6 – ADHD = 21, control = 32; and Grade 7 – ADHD = 13, control = 11.

The participants were classified according to subtype: ADHD-HI (*n* = 19, 12%), ADHD-PI (*n* = 81, 51%) and ADHD-C (*n* = 60, 37%).

Hand dominance: Of the ADHD group 146 (91%) were right-handed, while 14 (9%) were left-handed. The control group consisted of 149 (93%) children who were right-handed and 11 (7%) who were left-handed. The effect of hand dominance could not be included in the analysis because there were too few left-handers, resulting in empty cells when subtype, age and gender were taken into account. Therefore the results of each hand were analysed separately.

Table 1 presents the descriptive statistics and MANOVA results. The minimum statistical value was set at *p* < 0.05.

**TABLE 1:** Descriptive statistics and multivariate analysis of variance results for two motor tasks.

Variable	*n*	%	F	M	Age	Dominant hand	*p*	Non-dominant hand	*p*
**Pegboard**									
ADHD-HI	19	1	13	6	10.68 ± 1.49	91.42 ± 14.46	0.450	98.53 ± 40.67	0.430
ADHD-PI	81	51	40	41	10.40 ± 1.70	93.64 ± 34.49	0.001*	98.24 ± 23.29	0.011*
ADHD-C	60	37	27	33	10.47 ± 2.07	94.93 ± 24.80	0.002*	100.60 ± 23.56	0.004*
Control	160	100	80	80	10.49 ± 1.80	79.53 ± 26.31	-	88.22 ± 22.22	-
**Maze**									
ADHD-HI	19	12	13	6	10.68 ± 1.49	21.21 ± 12.27	0.330	26.47 ± 10.65	0.210
ADHD-PI	81	51	40	41	10.40 ± 1.70	25.87 ± 15.64	0.850	32.75 ± 22.52	0.960
ADHD-C	60	37	27	33	10.47 ± 2.07	31.28 ± 30.14	0.150	37.33 ± 33.37	0.130
Control	160	100	80	80	10.49 ± 1.80	26.43 ± 22.25	-	32.63 ± 22.47	-

ADHD, attention deficit hyperactivity disorder; HI, hyperactivity and impulsiveness subtype; PI, predominantly inattentive subtype; C, combined subtype; F, female; M, male.

* *p* < 0.05

On the Grooved Pegboard test, the MANOVA showed no main significant differences or interaction effects for gender and age for either the dominant or the non-dominant hand. Therefore, only the ADHD subtypes were further analysed. There were significant differences between ADHD subtypes in the performance of the dominant hand, *F*(2, 297) = 8.48, *p <* 0.001. Post-hoc analysis revealed a statistically significant difference between the ADHD-PI (*p* = 0.001) and ADHD-C (*p* = 0.002) and the control group only. These groups took longer to complete the task with their dominant hand. There was no statistical difference between ADHD-HI and the control group when using the dominant hand. There were also statistically significant differences in the performance of the non-dominant hand among the ADHD subtypes, *F*(2, 297) = 5.93, (*p* < 0.001). Post-hoc analysis (Bonferroni) further showed that the ADHD-PI (*p* = 0.011) and ADHD-C (*p* = 0.004) subtypes differed significantly from the control group in that the two ADHD groups took significantly longer to complete the task. There was no statistical difference when the ADHD-HI subtype was compared with the control group.

On the Maze Coordination Task, age had an effect on performance with both the dominant and non-dominant hands (*p* = 0.004). There were no effects for gender. The MANOVA indicated significant differences in performance for dominant hand, *F*(2, 297) = 6.05, *p* < 0.001; post-hoc analysis, however, revealed that this was because of the interacting effect of age, with the performance of the younger group significantly poorer than that of the older group. The difference was between the age groups only and not between the subtypes. The difference between the ADHD groups and the controls was not statistically significant, and there was no statistically significant difference between the subtypes in performance of the non-dominant hand, *F*(2, 297) = 2.15, *p* = 0.093.

## Discussion

The results of the study show that children with ADHD, especially the ADHD-PI and ADHD-C subtypes, were more impaired on the Grooved Pegboard Task, which measures distal, complex fine motor coordination and psychomotor speed, than on the Maze Coordination Task, which measures tactual coordination skills and motor planning.^44^ The poorer performance on the Grooved Pegboard of the ADHD-PI and ADHD-C subtypes suggest that their eye-hand coordination is impaired when motor speed is required,^39,45^ possibly because of problems with attention as the underlying manifestation of the motor skills deficit.^18,46^ This finding is consistent with most other studies,^15,39,47^ which found that the most pronounced impairment of motor functioning was in children with symptoms of ADHD-PI and ADHD-C subtypes and that inattention may affect motor skills.^3,48,49^ These studies compared motor problems in children with ADHD with neurotypical controls, and the conclusion reached was that difficulties in gross and fine motor skills were all related to symptoms of intention, not hyperactivity or impulsiveness.

The studies conducted by Ghanizadeh^48,50^ implied that fine motor skills of writing were predicted by the severity of symptoms of inattention. Motor responses require attention to a target and attention during the response. If the target is not properly attended to, it will affect the subsequent motor planning and consequent performance. Additionally, when the target is not noticed in time, it can reduce the time remaining for motor preparation and accordingly affect the performance.^13^ This may explain why the Grooved Pegboard, a timed task, discriminated better between children with ADHD and the neurotypical controls than the Maze Coordination Task, which was not timed and measured only motor planning and executions.

Not all studies on motor deficiencies in ADHD have found fine motor impairment to be limited to inattention. Scharoun et al.^51^ reported that all children with ADHD performed fine motor tasks more slowly than children without ADHD. Most studies, however, found that the ADHD-HI subtype was least affected.^3,39,52^ The conclusion drawn by these authors was that, unlike inattention, hyperactivity and impulsiveness was not a good predictor of deficiencies in fine motor performance in children with ADHD.

Our study also showed that age influenced Maze Coordination Task performance with both the dominant and non-dominant hands. These results are similar to the findings of Meyer and Sagvolden,^39^ who found that age had the most pronounced effect on the Maze Coordination Task, likely because of the maturation effect. Younger children with and without ADHD have poorer motor skills, visual planning and visual perceptual abilities than their older counterparts. Therefore, the task might have been too complicated for the younger participants.^39^ Meyer and her colleagues in a similar study conducted in 2006 among different cultures in Limpopo had comparable findings to our study. One can debate that a replication was necessary after a decade in an area where technology and educational facilities have improved. However, the similar results indicate that deficiency in fine motor skills is linked to ADHD symptomatology and is not affected by education and technology that has progressed over the past 10 years.

The cortical-striatal and cortical-cerebellar network is considered to consist of parallel circuits that underlie motor, cognitive and emotional behaviours.^53^ Therefore, it is evident that the underlying brain mechanisms are interrelated and that ADHD can be regarded as a multisystem developmental disorder with expressions in the cognitive, emotional and motor areas.^13,53^ Houwen et al.^54^ confirmed the relationship between motor performance and executive functions, finding that children who had motor coordination problems also performed poorly on tests of working memory and planning. Therefore, interventions for improving motor deficiencies may lead to improvement in cognitive and emotional functions.^13^

### Implication

Problems with attention are the underlying manifestation of the motor skills deficit. Motor problems lead to difficulties in everyday living, including academic performance, sports, play and self-esteem,^1,21,22,23^ and severely impact children’s daily lives. Fine motor problems are a strong predictor of a child’s popularity and self-esteem.^24^ Thus, poor motor performance may have an adverse effect on children’s self-esteem, anxiety and social functioning.^22,24^ Children with fine motor problems and ADHD are at risk for learning difficulties and deficient psychological adjustment.^55,56^

As noted by Gillberg et al.^10^ and Sergeant et al.,^30^ motor problems are not usually part of assessments for ADHD or incorporated into intervention programmes. It is vital that fine motor functions be part of evaluations for ADHD and included in general intervention methods for the treatment of children with ADHD and fine motor problems. Early detection of fine motor problems and identification of ADHD subtypes will help clinicians to recognise children at risk, who then can be referred to appropriate mental health workers for pharmacological treatment and/or behavioural modification intervention.

The DSM-5 focuses mainly on DCD as a separate neurodevelopmental disorder, while the fine motor skill deficiencies related to ADHD are not documented in the DSM-5.^2^ More research is needed to establish whether deficiencies in motor skills are usually comorbid with ADHD, or even can be regarded as diagnostic criteria for the disorder, or be considered as co-occurring pure DCD as stipulated in the DSM-5.

### Limitations

The sample does not represent the general population of primary school children in Limpopo Province as it was limited to Sepedi-speaking children in a particular municipal area. Owing to the homogeneity of the sample, socio-cultural differences could not be indicated. Future studies should include Sepedi along with other language groups. Another limitation was the small size of the ADHD-HI group, which may not represent all individuals with ADHD-HI. It may also be a limiting factor to compare 6-year-olds with 14-year-olds, who may already have entered puberty. Future studies should examine groups of children of the same age in regard to fine motor deficits and ADHD subtypes.

## Conclusion

Our study showed that children with ADHD have poorer fine motor skills than typically neuro-developing children. The performance of the children with ADHD showed deficiencies in distal, complex, fine motor coordination and psychomotor speed, as measured by the Grooved Pegboard Task. The results suggest that fine motor difficulties affect mainly the predominantly inattentive and combined ADHD presentations. Given the relationship between motor problems, especially handwriting, and academic performance, assessment of fine motor skills should be part of the ADHD diagnostic process. The result of this and other studies show that the Grooved Pegboard Task can be used as a valid instrument to detect motor deficiencies in children with ADHD.
